# Pathogenic LDLR Variants (c.103 C>T and c.2416dup) in ligand-binding and cytosolic domains in Saudi familial hypercholesterolemia: Molecular characterization and computational insights

**DOI:** 10.1016/j.csbj.2025.08.029

**Published:** 2025-08-25

**Authors:** Hadiah Bassam Al Mahdi, Noor Ahmad Shaik, Babajan Banaganapalli, Sherif Edris, Rawabi Zahed, Hanan Abdelhalim ElSokary, Hussam Daghistani, Yousef Almoghrabi, Safa Bayashut, Alaa Y. Edrees, Abdulrahman Mujalli, Eman Alefishat, Ramu Elango, Zuhier Awan

**Affiliations:** a Department of Biological Sciences, Faculty of Science, King Abdulaziz University, Jeddah, Saudi Arabia; b Department of Genetic Medicine, Faculty of Medicine, King Abdulaziz University, Jeddah, Saudi Arabia; c Princess Al-Jawhara Al-Brahim Centre of Excellence in Research of Hereditary Disorders, King Abdulaziz University, Jeddah, Saudi Arabia; d Department of Clinical Biochemistry, Faculty of Medicine, King Abdulaziz University, Jeddah, Saudi Arabia; e Department of Research and Development, Al Borg Diagnostics, Jeddah, Saudi Arabia; f Department of Clinical Pharmacology, Faculty of Medicine, Khalifa University, United Arab Emirates; g Regenerative Medicine Unit, King Fahd Medical Research Centr, King Abdulaziz University, Jeddah, Saudi Arabia; h Department of Clinical Laboratory Sciences, Faculty of Applied Medical Sciences, Umm Al-Qura University, Al Abdeyah, Makkah, Saudi Arabia

**Keywords:** Familial Hypercholesterolemia, LDLR gene, Whole Exome Sequencing, 3D structure, Molecular Docking, Molecular Dynamics Simulation

## Abstract

Familial hypercholesterolemia (FH) results in elevated levels of LDL-C, increasing the risk of developing cardiovascular disease. This study aims to identify genetic causes and examine the connection between genetic variants and the resulting genotype-protein phenotype in Saudi FH patients. Whole-exome sequencing (WES) and Sanger sequencing were employed to detect causative variants in affected Saudi FH families and their healthy relatives. Computational tools, including RNA stability analysis, molecular dynamics simulations, and molecular docking were used to assess the impact of these variants on mRNA stability and protein structure, particularly LDLR-LDLRAP1 interactions. WES identified two pathogenic variants in the LDLR gene in two Saudi FH families: c.103 C>T p.(Gln35Ter) and c.2416dup p.(Val806GlyfsTer11), both absent in healthy relatives and regional databases. The c.103 C>T variant alters the secondary RNA structure of LDLR, potentially affecting its stability and function. The c.2416dupG variant truncates the LDLR cytoplasmic tail, disrupting the NPXY–LDLRAP1 interaction and impairing receptor internalization. Molecular dynamics simulations using Desmond revealed increased structural flexibility and altered interaction dynamics in the LDLR protein due to the c.2416dup variant, suggesting further impacts on the protein’s functional integrity. In conclusion, this study identifies rare pathogenic variants c.2416dup and c.103 C>T in *LDLR* in extended Saudi Arabian families. It demonstrates the integration of bioinformatics methods with sequencing data to characterize and elucidate the pathogenic effects of genetic variants, providing comprehensive insights into the intricate interplay between LDLR genetic variants and their molecular impacts in FH patients.

## Graphical Abstract

**Figure:**
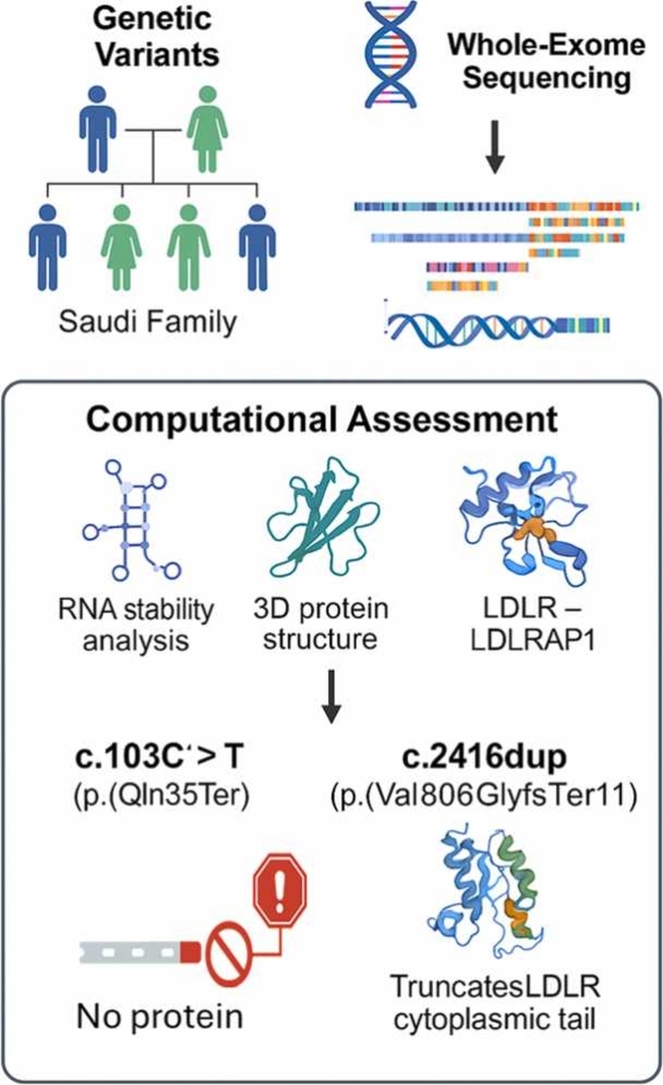

## 1 Introduction

Familial hypercholesterolemia (OMIM #143890) is a metabolic disorder that results from genetic defects leading to significantly elevated levels of low-density lipoprotein cholesterol (LDL-C) throughout a person's lifetime. The elevated LDL-C levels result in more than a 20-fold increase in the risk of premature cardiovascular disease (CVD) [1]. Moreover, FH manifests clinically in two principal forms: heterozygous FH (HeFH), where LDL-C levels are 2–3 times higher (190–390 mg/dL), and homozygous FH (HoFH), where LDL-C levels are 6–10 times higher (650–1000 mg/dL) than normal. The elevated LDL-C levels lead to the formation of atherosclerotic plaques in the coronary arteries and proximal aorta at an early age [2], and individuals with HoFH experience early onset CVD and premature Coronary heart disease (CHD) events in their mid-20s. FH is usually diagnosed based on the patient's lipid levels, age, family history, and genetic diagnosis [3].

FH can occur in either an autosomal dominant form, caused by monoallelic mutations in *LDLR, APOB*, or *PCSK9*, or an autosomal recessive form, due to bi-allelic mutations in the *LDLRAP1* gene [4]. Low-density lipoprotein receptor (LDLR) is a crucial membrane receptor that plays a key role in cholesterol homeostasis by mediating the endocytosis of cholesterol-rich low-density lipoproteins (LDL) from the bloodstream. Unfortunately, many patients with FH remain undiagnosed until after their first coronary heart disease (CHD) event. However, early genetic diagnosis and treatment can significantly improve life expectancy. Recent epidemiological studies have reported a high prevalence of FH in the adult population of the Gulf region, including Saudi Arabia, with a prevalence rate of 1 in 112 [5]. This is higher than the worldwide prevalence rate of 1 in 250–500 [6], [7]. The high prevalence of FH in the Arab population could be attributed to a high rate of consanguinity or founder effects of disease causative variants. Nonetheless, data on the genetic diagnosis of FH in the Arabian Peninsula remains limited [8], [9]. Therefore, raising awareness of FH and providing genetic testing and counseling to individuals with a family history or elevated cholesterol levels is crucial to facilitate early diagnosis, treatment, and improved patient outcomes.

Family-based genetic analysis, especially that involving family, is crucial for identifying and/or confirming rare causal genetic variants [10], [11]. However, next-generation sequencing approaches, such as whole exome sequencing (WES), generate millions of short-read sequence data in a much shorter time at a much lower cost. Different bioinformatics methods are utilized while searching for single nucleotide variations (SNVs) or INDELs in WES sequence data after performing quality control, trimming, and aligning the raw reads to a high-quality reference genome with annotation of the variants [12].

Bioinformatics-based molecular modeling and biophysical characterization of disease-causing variants are attracting considerable attention owing to their potential in developing genomic medicine [13]. Bioinformatics methods have often been shown to act as a potential pre-laboratory approach to assess the variant-induced impact on mutant proteins before undertaking technically challenging and expensive in vitro and in vivo studies [14], [15]. Although many FH variants have been reported in different ethnic groups, detailed bioinformatic characterization of those variants is rarely reported [16], [17]. Therefore, this study aimed to identify the genetic causes of FH in Saudi patients and to decipher the genotype-protein phenotype relationship of them using different bioinformatic methods.

## 2 Methodology

### 2.1 Family recruitment and clinical investigation

This study obtained ethical approval from the Biomedical Ethics and Research Committee at King Abdulaziz University, Jeddah, Saudi Arabia (reference number 220–22). For this study, families were enrolled in the Genetic Dyslipidemia and Familial Hypercholesterolemia Clinic at King Abdulaziz University Hospital, Jeddah, Saudi Arabia from August 2022 to May 2023. Individuals in the family were investigated with a battery of laboratory investigations for various biochemical parameters, such as plasma lipid profile (LDLC, HDL-C, Triglyceride, and Total Cholesterol). Blood glucose levels, thyroid, and liver function tests were also performed to exclude secondary causes of hypercholesterolemia. All biochemical tests were performed on individuals prior to treatment, using enzymatic assays conducted on a semi-automated autoanalyzer. The physician followed up with the patients, interviewed them, obtained their family history, generated a pedigree, and recruited their remaining family members. Informed consent forms were obtained verbally from all study participants, followed by signing a written consent form prior to blood sampling and genetic testing. Participants under the age of 18 were accompanied by their parents or guardians, and consent was obtained from both the participant and their legal guardian. All patients met the FH diagnostic criteria of the Simon Broome Register and Dutch Lipid Clinic Network (DLCN) [18], [19]. The DLCN criteria use a combination of clinical and laboratory data, including family history, LDL cholesterol levels, and genetic testing, to identify individuals with FH, while the Simon Broome criteria rely on clinical data, such as the presence of tendon xanthomas and family history, to make a diagnosis of FH.

### 2.2 DNA isolation

Approximately 2 mL of whole EDTA peripheral blood samples were collected from all participants in the study. DNA was isolated using the QIAamp DNA Mini Kit (Qiagen, Alameda, CA, USA) according to the manufacturer’s instructions. The quality, quantity, and integrity of the DNA were evaluated using a Denovix DS-11 spectrophotometer and 1 % gel electrophoresis.

### 2.3 Whole exome sequencing

The index's genomic DNA (2 µg) was analyzed in exome libraries using SureSelect V6 library preparations (Agilent Technologies, USA). Subsequently, the NextSeq 6000 sequencer (Illumina, USA) was used to generate massive parallel short-read sequencing data (100x coverage). The reads were aligned with the Burrows-Wheeler aligner [20] and mapped using the hg19 genome database (UCSC Genome Browser). SAMtool was used to detect copy number variations or indels that were later compared against sequences in the dbSNP and 1000 Genomes Project databases.

### 2.4 Candidate variant selection

To identify potential disease-causing variants, we analyzed WES data focusing on FH candidate genes curated from the GeneCards Database (https://www.genecards.org/), including LDLR, APOB, PCSK9, LDLRAP1, and additional FH-related genes. Variants that did not meet specific criteria were excluded. In particular, we excluded variants with a read depth less than 25 and those that were not coding variants, including missense, frameshift, splicing, and stop-loss/stop-gain variants. From the remaining variants, we selected those with a minor allele frequency (MAF) of less than 1 % and a CADD score of greater than 20, a SIFT score of less than 0.05, and a Polyphen2 score of greater than 0.7. Any variants that did not meet these criteria were excluded. To validate the allelic frequency of the remaining variants, we used several databases, including the Exome Aggregation Consortium (ExAC), the Greater Middle East (GME) Variome Project, the Allele Frequency Aggregator (ALFA) project, the Saudi KAIMRC database (https://kaimrc.ksau-hs.edu.sa/), and the Genome Aggregation Database (https://gnomad.broadinstitute.org/). Additionally, we determined the clinical pathogenicity of the variants according to ACMG criteria [21] using VarSome (https://VarSome.com).

### 2.5 Variant validation and familial segregation

To validate the potential candidate variant for FH identified in the WES data, Sanger sequencing was performed. This involved carrying out Polymerase Chain Reaction (PCR) using DreamTaq PCR Master Mix (catalog # K9021) and the VeritiPro 96-well thermal cycler from Applied Biosystems (Life Technologies, CA). The primer sequences used in the PCR reaction for family A (forward 5′ GCTGGTCTCGAACTCTTGACC 3′ and reverse 5′ ATTGTCCGCTGACATTCTGAA 3′) and family B (forward 5′ CCCATACCCCAGAGAGTCCA 3′ and reverse 5′ AGAATCGTGTCACAGGCCAG 3’). Both primers were designed with the assistance of Primer3Plus (https://www.bioinformatics.nl/cgi-bin/primer3plus/primer3plus.cgi). Following PCR amplification, cycle sequencing and Sanger sequencing were performed using the SeqStudio™ Genetic Analyzer (Life Technologies). Finally, the SnapGene version 6.0.2 software was utilized for alignment and identification of sequence variants.

### 2.6 RNA fold

The RNAfold web server [22] was utilized for predicting the secondary structures of single-stranded RNA sequences of *LDLR* gene variants, including both the wild type and targeted variant. Considering the limitation of 7500 nt per submission, we selected 1000 bp sequences for analysis, ensuring the variants were centered within the selected regions. For the c.103 C>T variant, the wild-type sequence was taken from chromosome 19:11210534–11211534, and the mutant sequence from the same region with the variant incorporated. For the c.2416dup variant, the wild-type sequence was taken from chromosome 19:11239715–11240715, and the mutant sequence from the same region with the variant incorporated. The sequences were submitted into the server, and we engaged the minimum free energy (MFE) and partition function methods were applied for structure prediction. Our methodology includes the consideration of dangling energies on both sides of a helix, using the Turner model (2004) RNA parameters, with energy parameters adapted to a physiological temperature of 37°C and a salt concentration of 1.021 Molar. The output, comprising interactive RNA secondary structure plots with reliability annotation and mountain plots, facilitates a detailed analysis and comparison of the structural alterations between the wild-type and mutant variants, thus aiding in understanding the potential functional impacts of these specific variants.

### 2.7 Protein modelling

The complete structure of the LDLR protein has not been experimentally resolved. To overcome this, the full-length model of LDLR protein was predicted by Alphafold v2.1.0, a machine-learning method [23]. This computational method utilizes a deep learning algorithm that combines biological and physical characteristics of protein structure by incorporating multiple sequence alignments. Finally, the simulated LDLR structure was validated with the help of Procheck tool. Furthermore, the PDBSUM server was used to evaluate the secondary structural components of the LDLR protein.

### 2.8 Topological analysis of LDLR Protein

To detect the localization of LDLR protein in the membrane region, we used Protter (http://wlab.ethz.ch/protter/start/), an open webserver that integrates protein sequence features and transmembrane topology into illustrations. Protter not only detects the membrane-spanning regions, but also identifies PTMs, variants, disulfide bonds, signal peptides, N-terms (UniProt), and TMRs (UniProt) in the protein, ID: P01130.

### 2.9 Molecular docking of LDLR-LDLRAP1

The ClusPro molecular docking tool (https://cluspro.org) was used to examine the protein-protein interaction between the molecules of LDLR and LDLRAP1. This webserver supports PDB format files of both receptor and ligand files. The server uses the PIPER docking algorithm, which evaluates the ligand positions in various rotations while conserving a fixed position for the center of mass of the receptor molecule. A rigid pairwise docking potential approach, based on quick Fourier transformations, is used by the ClusPro service to provide protein-protein interaction conformations with a low energy score. Using the default docking setting, the LDLRAP1 ligand rotations were adjusted to 70 K at each grid point in three directions (x, y, and z) around the LDLR receptor’s 3D grid-spacing of 1.0. The top 10 clusters were chosen from the 1000 best energy conformation outputs. The pose in each cluster with the lowest energy is the first pose to be clustered within 9 Å. Stable complexes were improved using Monte Carlo simulations. The molecular interactions (hydrophobic contacts, hydrogen bonds, and complexes involving receptors and ligands) in docking complexes were identified using the Protein Interaction Z-Score Assessment (PIZSA) program.

### 2.10 Molecular Dynamics Simulations

We applied molecular dynamics (MD) simulations using Desmond to study the dynamic behavior of wild-type and mutant variants of the Low-Density Lipoprotein Receptor (*LDLR*) over a 500-nanosecond period. For the wild-type receptor, we used an 860-amino-acid chain was used, whereas for the mutant, an 805-amino-acid chain was utilized. The OPLS3e forcefield, a computational model designed to simulate molecular interactions, guided the simulations [24]. The simulations began with energy minimization in a vacuum using the steepest descent approach. Next, the solution was achieved using a periodic cubic box filled with SPC water molecules. A buffer distance of 10 Å was maintained from the protein surface, resulting in a simulation box of approximately 120 × 120 × 120 Å³ . The solvated system contained ∼37,500 water molecules. To maintain electroneutrality and mimic physiological ionic strength, Na⁺ and Cl⁻ counterions were added to a final concentration of 0.15 M, including 18 Na⁺ and 16 Cl⁻ ions in the wild-type system (similar numbers for the mutant) [25]. During the 500-nanosecond simulation, the system was equilibrated in an NPT ensemble to keep temperature and pressure constant.

We analyzed structural changes, stability, and flexibility in the LDLR using tools for root-mean-square deviation (RMSD), root-mean-square fluctuation (RMSF), and secondary structure calculations in Desmond. Principal Component Analysis (PCA) and free energy landscape calculations were also performed to explore receptor motion patterns and energetically preferred states. This comprehensive approach allowed us to capture the receptor’s morphology, flexibility, and interactions with the surrounding environment, providing insights into its three-dimensional dynamics.

## 3 Results

### 3.1 Clinical phenotype and pedigree analysis

**Fig. 1:**
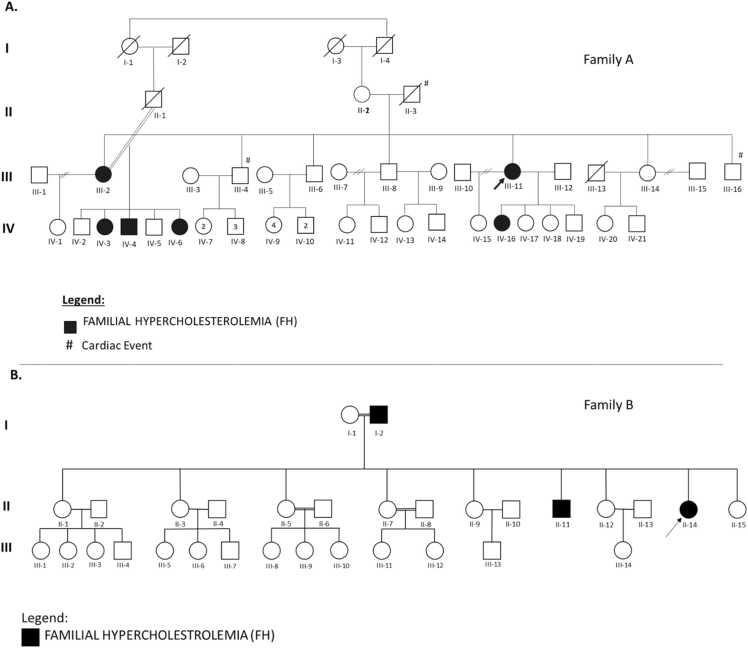
A pedigree chart of Saudi families with FH patients: (A) Family A; (B) Family B. The arrow indicates the proband (index case) in each pedigree. Filled symbols represent individuals clinically diagnosed with FH. Unfilled symbols denote individuals with normal lipid profiles or with unknown clinical and/or genetic status.

In this study, we initially selected two patients clinically diagnosed with FH as index cases for familial investigation. Both individuals exhibited markedly elevated LDL-C levels and a positive family history of hypercholesterolemia or a family history of premature cardiac events. Their DLCN scores ranged from 6 to 7, indicating a probable FH diagnosis. Subsequently, a total of 21 family members from both index cases were recruited for biochemical assessment (Table 1 and Supplementary Table 1). Pedigree analysis (Fig. 1) suggests an autosomal dominant mode of inheritance for FH in both families.

**Table 1:** Demographic data, clinical phenotype, and vital signs of the family members, including proband and other affected family members.

**Family A**								
Patients ID		Age	Gender	Diagnosis	LDL-C	BMI	Blood Pressure	Smoking
III−11	Proband	43	Female	FH	360	35.2	96/72	No
II−2	Mother	75	Female	Diabetic	107	44	NA	No
IV−15	Daughter	22	Female	Normal	90	17.5	NA	No
IV−16	Daughter	20	Female	FH	296	21.1	122/75	No
IV−17	Daughter	19	Female	Normal	86	22.7	99/77	No
IV−18	Daughter	15	Female	Normal	85	22.4	NA	No
III−2	Sister	53	Female	FH	409	27.7	110/65	No
IV−2	Nephew	28	Male	Hypercholesterolemia	134	27.5	NA	Yes
IV−3	Niece	26	Female	FH	386	22.8	84/61	No
IV−4	Nephew	25	Male	FH	369	25.1	102/69	Yes
IV−5	Nephew	23	Male	Hypercholesterolemia	143	46.5	115/84	Yes
IV−6	Niece	19	Female	FH	295	26.9	111/66	No

**Family B**								
Patients ID		Age	Gender	Clinical Phenotype		BMI	Blood Pressure	Smoking
II−14	Proband	22	Female	FH	286	32.4	118/82	No
I−2	Father	65	Male	FH	_	22.3	125/67	yes
I−1	Mother	59	Female	Normal	71	24.3	117/75	No
II−1	Sister	40	Female	Normal	88	26.3	100/65	No
II−7	Sister	34	Female	Diabetic Type 1	113	23.3	105/65	No
II−9	Sister	31	Female	Hypercholesterolemia	137	25.3	166/115	No
II−11	Brother	28	Male	FH	516	23.4	109/74	Yes
II−12	Sister	24	Female	Hypercholesterolemia	143	36.1	103/77	No
II−15	Sister	16	Female	Normal	118	21.8	117/76	No

**Family A:** The proband (III-11), a 43-year-old Saudi female from the western region of Saudi Arabia, with a mother of Turkish ethnicity, presents with severe hypercholesterolemia (LDL-C: 445 mg/dL). The pedigree analysis (Fig. 1) shows that two of her brothers (III-4 and III-16) experienced cardiac events at a young age (30 years old), and both have a history of cardiac events. Three of her four daughters from two marriages (IV-15, IV-17, and IV-18) have normal LDL-C levels, while the fourth daughter (IV-16) exhibits elevated LDL-C (296 mg/dL). Notably, the proband's sister (III-2), diagnosed with FH, has three affected children (IV-3, IV-4, and IV-6) with significantly elevated LDL-C levels (386 mg/dL, 369 mg/dL, and 295 mg/dL, respectively); IV-3, in particular, exhibited tendon xanthomas. While individuals II-2, IV-2, and IV-5 have higher LDL-C levels (100–190 mg/dL) compared to the controls, these levels fall below the diagnostic threshold for FH (>190 mg/dL). Additionally, several family members (III-4, III-6, III-8, III-14, and III-16) reported a diagnosis of hypercholesterolemia based on previous medical records but declined participation in genetic screening (Table 1 and Supplementary Table 1).

**Family B:** The proband (II-14), a 22-year-old Saudi female also from the western region, presents with severe hypercholesterolemia (LDL-C: 286 mg/dL). Her brother (II-11) is also affected with significantly elevated LDL-C (516 mg/dL), while her father (I-2) presented with a notable clinical sign of corneal arcus. Two sisters (II-2 and II-7) have optimal LDL-C levels, while the remaining siblings (II-9 and II-12) show elevated LDL-C (100–190 mg/dL) but do not meet the diagnostic criteria for FH (>190 mg/dL) (Table 1 and Supplementary Table 1). The demographic information, clinical phenotypes, vital signs, and biochemical laboratory findings for all participants are detailed in Table 1 and Supplementary Table 1. Biochemical measurements were obtained before the initiation of any lipid-lowering medications. Following diagnosis and genetic confirmation of FH, the individuals were initiated on lipid-lowering therapy, including Repatha® (evolocumab) and statins, with dosages individualized based on their cardiovascular risk profiles and LDL-C levels.

Based on biochemical findings from 21 individuals in both families, a total of nine individuals were clinically diagnosed with FH with DLCN scores between 6 and 7, six individuals presented with normal lipid profiles, 4 were identified with non-genetic hypercholesterolemia, and 2 individuals were diabetic without hypercholesterolemia. These classifications were further used to support familial segregation analysis and confirm the clinical investigation.

### 3.2 Whole exome sequencing and variant filtering

**Fig. 2:**
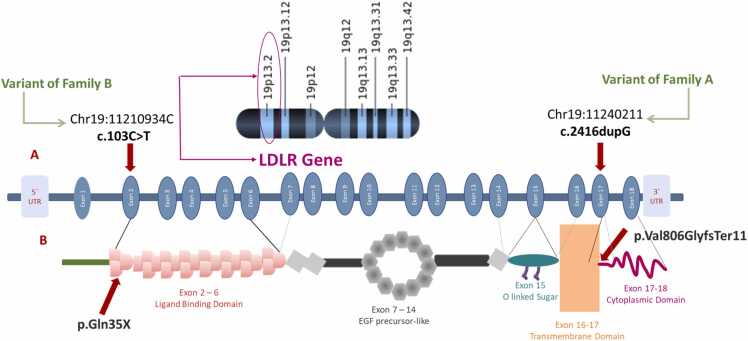
Mapping of Pathogenic LDLR Variants and Protein Domains: Schematic illustration of the LDLR gene and protein structure, showing the location and domain context of the two pathogenic variants identified in this study. (A) Chromosomal localization of the LDLR gene at 19p13.2 and exon-intron structure. The c.103 C>T (p. Gln35X) variant of family B is in exon 2, within the ligand-binding domain (exons 2–6), while the c.2416dupG (p.Val806GlyfsTer11) variant of family A, is in exon 17, affecting the transmembrane (exons 16–17) and cytoplasmic domains (exons 17–18). (B) Protein domain architecture, including the ligand-binding domain, EGF precursor-like domain, O-linked sugar region, transmembrane domain, and cytoplasmic tail, with respective variant impacts indicated by red arrows.

Whole-exome sequencing identified a significant number of variants in the probands from both families. Family A harbored 142,350 annotated variants, while family B had 136,181 variants. By focusing on known genes associated with Familial Hypercholesterolemia (FH), we pinpointed variants within the *LDLR* gene in both families according to the GRCh37/hg19 human genome assembly (Fig. 2). The identified variants were subjected to further analysis for familial segregation.

In family A, the variant NC_000019.9(NM_000527.5):c.2416dup (rs773618064), located in exon 17 of chromosome 19:11240211 introduces a guanine nucleotide (G) within a repetitive region of five guanines. This insertion creates a frameshift variant, altering the protein's amino acid sequence at position 806 p.(Val806GlyfsTer11). In family B, the variant NC_000019.9(NM_000527.5):c.103 C>T (rs879254408) located in exon 2 of chromosome 19:11210934 substitutes cytosine with thymine (C>T). This substitution results in a stop gain variant, prematurely truncating the protein sequence at position 35 p.(Gln35Ter).

The *LDLR* variants c.2416dup and c.103 C>T have been classified as pathogenic according to the ACMG/AMP guidelines, as supported by the VarSome database and documented in ClinVar. Specifically, c.2416dup is associated with ClinVar accessions RCV000238458.12, RCV000486216.6, and RCV000780378.8; while c.103 C>T is linked to RCV000237937.2 and RCV001036722.6.

According to ClinVar, both variants are consistently classified as pathogenic for familial hypercholesterolemia and fulfill the ACMG/AMP criteria. Particularly, NM_000527.5:c.2416dup (p.Val806GlyfsTer11) corresponds to Variation ID: 252330, and NM_000527.5:c.103 C>T (p.Gln35Ter) corresponds to Variation ID: 251021.In the gnomAD database, the variant c.2416dup shows a low minor allele frequency (MAF = 0.0000239), while c.103 C>T was not reported. Additionally, neither variant was found in regional population databases such as ALFA, GME, or KAIMRC. However, studies from other countries have documented the presence of c.2416dup variant (Table 2).

**Table 2:** Review of the clinical studies, which reported c.2416dup in different populations.

**Population**	**# of Patients**	**Sex**	**Zygosity**	**Clinical phenotype**	**Reference**
American	6	NA	NA	NA	[50]
American	NA	NA	NA	NA	[76]
Brazilian	1	NA	Heterozygous	FH	[77]
British	1	NA	Heterozygous	FH, myocardial infarction	[43]
Chinese	NA	NA	Heterozygous	NA	[78]
Chinese	1	Male	Heterozygous	FH	[79]
Czech	1	NA	NA	NA	[80]
Hungarian	2	2 Females	Heterozygous	FH, coronary artery disease, and xanthelasma	[81]
Indian	3	NA	Heterozygous	FH	[36]
Indian	11	6 Females, 5 Male	Heterozygous	FH, corneal arcus, and MI at age 37	[44]
Indian	1	2 Females 1 Male	2 Heterozygous and Homozygous	Tendon xanthomas, corneal arcus, and family history of CAD	[46]
Iranian	2	NA	Homozygous	NA	[30]
Japanese	4	NA	NA	NA	[82]
Japanese	NA	NA	NA	NA	[83]
Japanese	3	NA, Male, Female	1 Heterozygous, Compound Heterozygous	NA, Tendon xanthomas	[45]
Norwegian	NA	NA	NA	NA	[84]
Pakistani	10	5 Females, 5 Males	9 Heterozygous and 1 Homozygous	NA	[85]
Poland	5	3 Females, 2 Males	Heterozygous	FH	[86]
Russian	2	NA	Heterozygous	FH	[87]
Russian	1	NA	NA	NA	[88]
Swedish	1	NA	Heterozygous	FH	[89]
Swedish	NA	NA	NA	NA	[49]
Turkish	NA	NA	Heterozygous	FH	[29]

### 3.3 Familial segregation

**Fig. 3:**
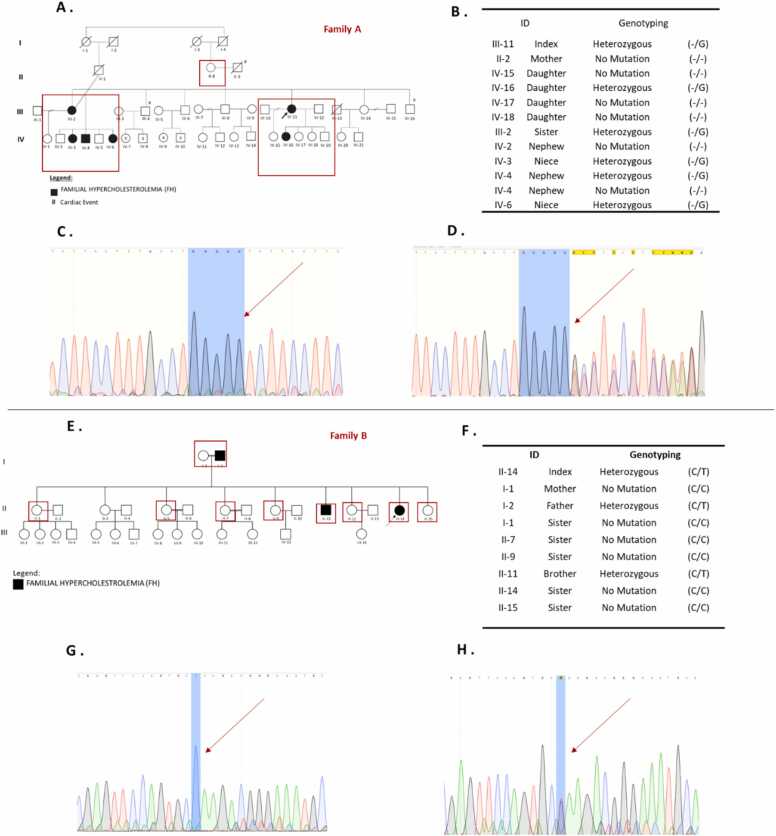
Familial segregation and Sanger sequencing analysis in Families A and B. (A) Pedigree of Family A, with red boxes highlighting individuals who underwent genetic testing. (B) Genotyping results of Family A members showing the presence or absence of the c.2416dup variant. (C) Representative chromatogram showing a wildtype sequence of (no duplication) at the *c.2416* position. (D) Chromatogram indicating a heterozygous frameshift variant (c.2416dup), demonstrated by overlapping peaks following the duplication site. The blue shading indicates the homopolymeric G-rich region, and the red arrow marks the exact site of insertion. (E) Pedigree of Family B, with red boxes marking individuals enrolled for genetic testing. (F) Genotyping results of Family B members regarding the c.103 C>T variant. (G) Chromatogram showing the wild-type allele (C) at position 103. (H) Chromatogram showing a heterozygous c.103 C>T nonsense variant, indicated by overlapping peaks at the mutation site (noted as "Y" for C/T heterozygosity).

Sanger sequencing confirmed that the *LDLR* c.2416dup and c.103 C>T variants in the index family exhibit an autosomal dominant mode of segregation. In family A, the proband (III-2) and her sister (III-11), carried heterozygous c.2416dup variants and transmitted them to their respective offspring (IV-3, IV-4, IV-6, and IV-16). This variant was absent from other tested family members (II-2, IV-2, IV-5, IV-15, IV-17, and IV-18). Based on pedigree analysis, the variant (c.2416dup) likely inherited from their deceased father (II-3), consistent with autosomal dominant inheritance. Similarly, in family B, the proband (II-14) and the brother (II-11) carried a heterozygous variant (c.103 C>T) inherited from their father (I-2) carried a heterozygous variant. On the other hand, it was absent among other members (I-1, II-1, II-7, II-9, II-12, and II-15). Sequence chromatograms for the wild-type and heterozygous variants (c.2416dup and c.103 C>T) are presented in Fig. 3.

### 3.4 RNA fold analysis

**Fig. 4:**
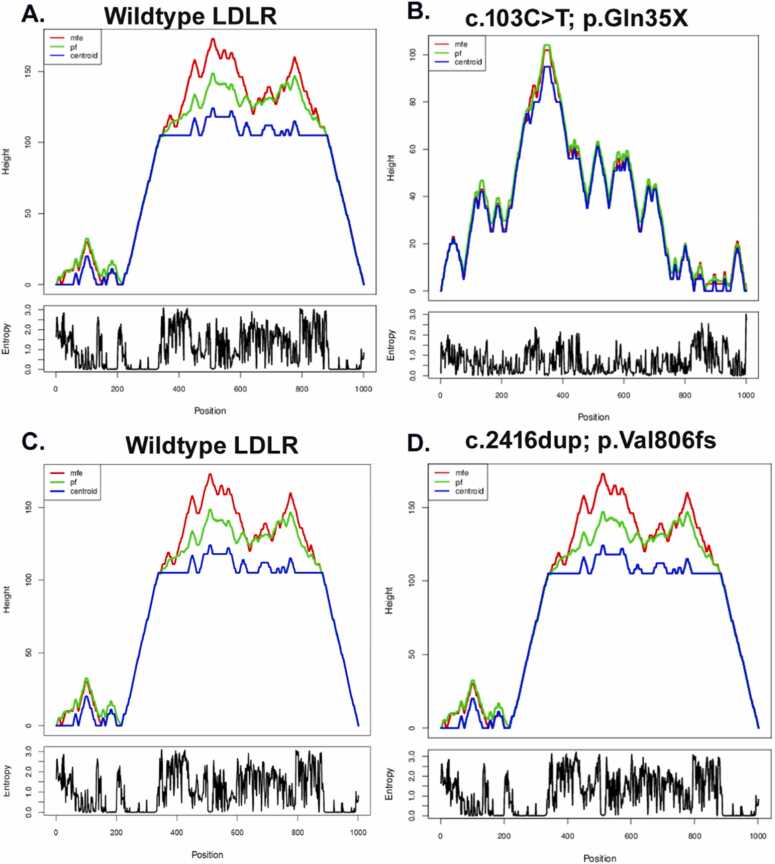
Comparative Analysis of LDLR Gene Variants and their Impact on RNA Structure: A) Illustrates the RNA secondary structure for the LDLR wildtype variant, emphasizing stability and predicted RNA interactions. B) Displays the centroid secondary structure and mountain plot for the LDLR mutant c.103 C>T p. (Gln35Ter), highlighting the variant's effect on secondary structure, including changes in base-pairing probabilities and the structural landscape. C-D) Details the centroid secondary structure and mountain plot for the LDLR wild and mutant c.2416dup p. (Val806GlyfsTer11), showcasing the profound structural alterations due to the duplication variant, as reflected in the variation of base-pairing probabilities and the overall structural configuration.

The RNAfold analysis provides a detailed comparison of the structural configurations between the wildtype and mutant versions of the LDLR RNA structure, as illustrated in Fig. 4. For the wildtype variant c.103 C>T, the analysis determined a minimum free energy (MFE) of −301.70 kcal/mol, indicative of a highly stable predicted secondary RNA structure. In contrast, the corresponding mutant variant revealed a marginally higher MFE of −299.40 kcal/mol, suggesting a decrease in structural stability which could potentially affect RNA functionality.

Furthermore, analysis of the c.2416dup; variant showed that the wildtype form possesses an MFE of −427.30 kcal/mol, while its mutant counterpart demonstrates a slight reduction in stability, with an MFE of −426.60 kcal/mol. A significant finding from the analysis is the increase in ensemble diversity for the mutants, with values of 153.08 kcal/mol for c.103 C>T and 244.54 kcal/mol for c.2416dup, highlighting enhanced structural variability introduced by these variants. The RNAfold analysis reveals significant structural differences between the wildtype and mutant variants of the *LDLR* gene. For the c.103 C>T variant, the differences are most pronounced in the 5′ region (nucleotides 1–100), where the wildtype RNA forms stable stem-loop structures, while the mutant RNA shows disruptions in these regions, leading to a more open conformation. For the c.2416dup variant, the differences are mainly observed in the 3′ region (nucleotides 700–800), where the wildtype RNA exhibits well-formed stem structures, whereas the mutant RNA has more extensive loop regions and fewer stable helices, resulting in a less compact structure (S3 Fig). This comprehensive examination underscores the critical role that specific variants can play in altering the RNA secondary structure and overall stability of the LDLR molecule.

### 3.5 Molecular modelling of LDLR

The full-length predicted LDLR protein was accessed from the AlphaFold2 database The partial X-ray structure of LDLR (255 A.A. to 651 A.A.) and the structure predicted by AlphaFold2 were nearly identical, with an overall root-mean-squared-deviation (RMSD) of less than 2 Å (S1 Fig). The predicted LDLR structure reports a low predicted average error < 5 Å (PAE, a per-residue pair distance score), a high predicted local distance difference test score of > 90 (pLDDT, a per residue 0–100 score) showing more confidence. According to the Procheck analysis, 98.6 % of the LDLR amino acids were present in the favored and allowed regions (S1 Fig). The PDBSUM server predicted the secondary structure of LDLR with 860 residues, which consists of 224 (26.0 %) strands, 98 (11.4 %) helices, and 538 (62.6 %) other components (S2 Fig). However, in truncated LDLR, the membrane-spanning H18 helix and C-terminal cytosolic (806–860 amino acids) regions were deleted.

### 3.6 LDLR structural localization

Protter analysis revealed three distinct cellular localizations for the LDLR protein: the extracellular area from 1 to 788 A.A (which includes the signal peptide domain from 1. A.A to 21 A.A); the transmembrane domain from 289 AA to 810 AA; and the cytosolic region from 811 A.A to 860 A.A. This analysis clearly demonstrated that the truncated LDLR lost not only its cytoplasmic region amino acids, but also a part of the transmembrane region (806–810 A.A) (S2 Fig)

### 3.7 Molecular docking analysis of LDLR with LDLRAP1

Molecular docking analysis was performed using the ClusPro program, where the LDLR was selected for docking with the LDLRAP1 protein. The resulting LDLR-LDLRAP1 complex exhibited hydrogen bond in (Asn825(A)-Lys87(B); Gly829(A)-Leu107(B); Asp853(A)-Ser81(B); Glu856-Thr105(B)) (**S2 Fig**). The cluster energy of the docking complex was −218.6 kJ/mol, indicating a stable interaction. The intracellular regulatory mechanism of LDLR-LDLRAP1 binding may be compromised in the truncated LDLR, as the loss of the C-terminal domain of LDLR could disrupt its interaction with LDLRAP1, potentially affecting LDLR recycling and degradation.

### 3.8 Molecular dynamics simulations

The wild-type LDLR protein had a reduced overall Root Mean Square Deviation (RMSD), indicating that its conformation remained stable throughout the simulation. The RMSD results exhibited fluctuation around an average of roughly 12 Å, suggesting a system that is in a state of equilibrium after 100 ns. The mutant LDLR exhibited an elevated RMSD that progressively decreased, reaching a maximum value of 10.5 Å. This indicates significant conformational alterations, most likely caused by the shortened structure. The examination of Root Mean Square Fluctuation (RMSF) yielded valuable insights into the protein's local dynamics. The mutant LDLR displayed heightened fluctuations, specifically in the N-terminal region (residues 1–550) and in close proximity to the truncation location, spanning from residues 750–805. These regions exhibited RMSF peaks that were notably greater than those observed in the same regions of the wild-type LDLR. In the wildtype protein, high RMSF values are predominantly located around residues 100–200, 300–400, and 700–800, likely corresponding to flexible loop regions and termini of secondary structure elements. The mutant protein shows significantly higher RMSF values in similar regions, particularly around residues 100–200 and 700–800, with an additional peak observed near the C-terminus. This increased fluctuation suggests that the variants have introduced greater flexibility and instability in these regions.

**Fig. 5:**
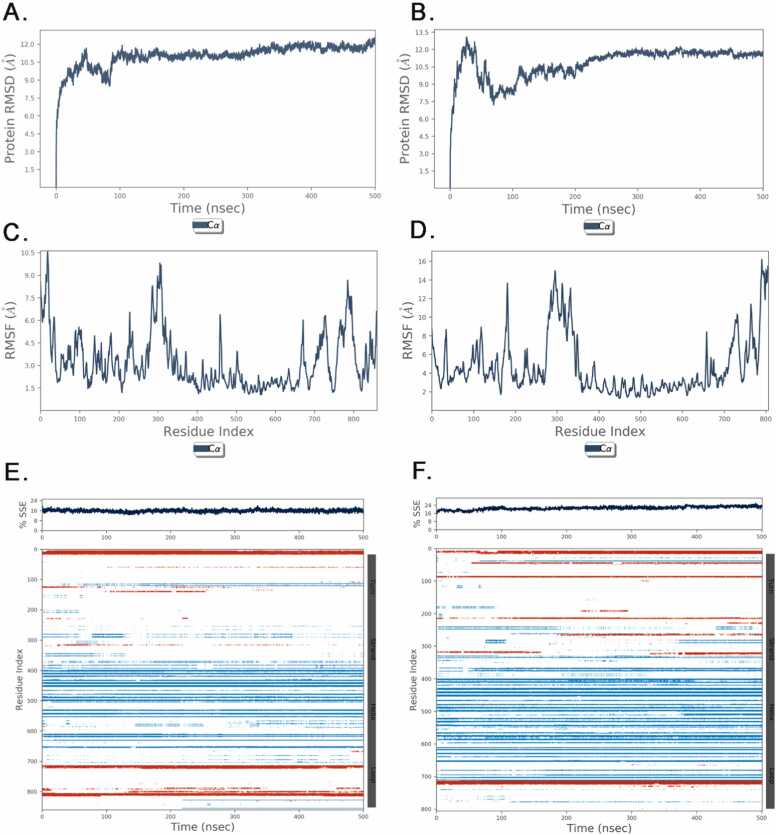
LDLR Dynamics: stability, flexibility, and variant Effects in simulation: (A) shows the Root Mean Square Deviation (RMSD) of the wildtype LDLR protein throughout a 500-nanosecond simulation run, proving the stability and steady structure of the protein. The relative RMSD of the mutant LDLR protein showed an indication of the structural deviations due to the truncation. (C) Wildtype and (D) mutant: LDLR Root Mean Square Fluctuation (RMSF) show that the amino acid residues within the protein structure are flexible. Time evolution of the predicted secondary structure elements (SSEs) for wild-type and mutant LDLR, i.e., Panel (E) and (F) indicate the more "natural" behaviour for α-helices (blue), beta-strands (red), and turns (light blue), loops (white/blank space) along the trajectory of the simulation.

This suggests an aberration in the stability of the receptor's ligand-binding domain. The analysis of the DSSP graphs indicates that the most significant changes in beta sheet content occur in the 3′ region (residues 700–800) of the LDLR protein. In the wildtype structure, this region forms stable beta sheets, which are disrupted in the mutant structure, increasing in loop and coil regions. The wildtype LDLR exhibited a stable protein conformation, as evidenced by its constant secondary structure consisting of roughly 4.83 % helix and 10.98 % strand composition. On the other hand, the mutant LDLR exhibited a significant reduction in helical content, reaching 4.62 %, while simultaneously experiencing an increase in strand composition, reaching 16.51 %. The truncation resulted in the absence of the helix creating the H18 segment (amino acids 806–860) in the C-terminal region, causing a significant shift. It is probable that this modification has an impact on the overall conformation and stability of the receptor (Fig. 5).

**Fig. 6:**
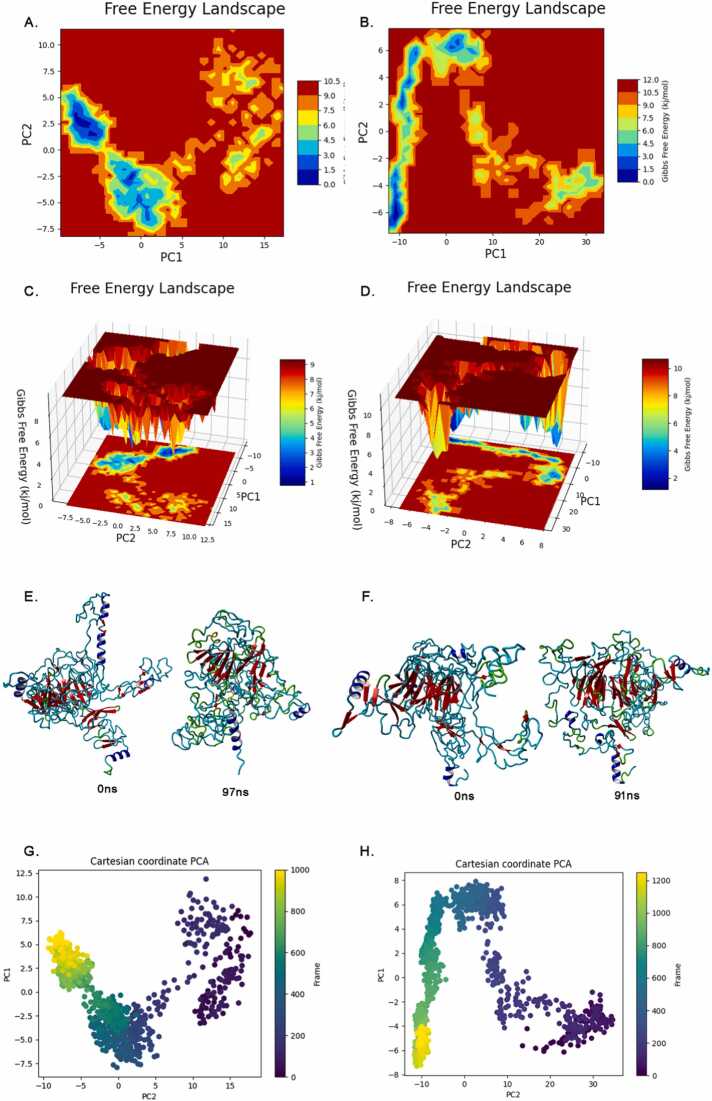
Comparative insights into LDLR: Free energy landscapes, temporal structural evolution, and PCA of wildtype vs. mutant forms. Panels (A) and (B) show the 2D free energy landscapes derived from principal components PC1 and PC2 for wildtype and mutant LDLR, respectively, illustrating the energy basins sampled during simulation. Panels (C) and (D) provide 3D representations of the same landscapes, highlighting the depth and spread of conformational states. Panels (E) and (F) display structural snapshots of LDLR at the start (0 ns) and at the time of reaching stable conformations (97 ns for wildtype, 91 ns for mutant). Panels (G) and (H) present the PCA projections of wildtype and mutant trajectories, respectively, with each point representing a frame colored by simulation time. The time interval between frames is 0.5 ns. Panel G includes 1000 frames (up to 500 ns), while panel H includes 1200 frames (up to 600 ns), reflecting the extended simulation of the mutant form to capture delayed convergence and increased structural flexibility.

From the molecular dynamics simulations conducted over 500 ns, we have gleaned notable insights into the free energy landscapes of both wild-type and mutant LDLR. For the wild-type LDLR, Free energy landscape is characterized by several favorable conformational states, with the lower Gibbs free energy. The landscape appears rugged with multiple minima, suggesting a variety of stable configurations that the wild-type LDLR can potentially adopt. The three-dimensional view of free energy landscape further clarifies the energy profile of the wild-type LDLR, showing pronounced energy wells that correlate with the stable conformations. The energy barriers visible between these wells provide an indication of the conformational transitions that the protein can undergo. The mutant LDLR displays a distinct energy landscape, with a redistribution of low-energy states compared to the wild-type. This suggests that the variant alters the protein's stability and shifts its preferred conformational states. The number and depth of the energy minima have been altered, indicating potential differences in the behaviour of the mutant protein. The three-dimensional free energy perspective variation in the landscape's topology due to the variant is more pronounced. The mutant LDLR displays a landscape with distinct energy barriers and wells, suggesting that its thermodynamic and conformational properties diverge substantially from those of the wild-type (Fig. 6).

Principal Component Analysis (PCA) was performed to identify the dominant motions and structural changes in the LDLR protein. The analysis revealed that the first two principal components (PC1 and PC2) had significant eigenvalues, capturing over 70 % of the total variance in the dataset. Specifically, PC1 accounted for 45 % of the variance and PC2 for 25 %. This indicates that these PCs represent the most dominant motions in the LDLR protein. Along with the first principal component (PC1), we observed significant conformational changes involving the opening and closing motions of the N-terminal domain and the central core of the protein. Along the second principal component (PC2), the primary structural changes involved the relative movement between the N-terminal and C-terminal regions, leading to changes in the overall compactness of the protein structure. These motions indicate that the LDLR protein undergoes significant conformational flexibility, which is further amplified in the mutant forms. The eigenvectors were calculated from a combined ensemble of both wild-type and mutant LDLR proteins to ensure comparability of the landscapes. This approach allows for the identification of common motions and structural changes across both wild-type and mutant forms. The combined ensemble was used to generate a covariance matrix, from which the eigenvectors were derived, representing the principal components of the motion (Fig. 6). The PCA plot shows a clear separation of trajectory frames across time points, indicating that the system may not have reached equilibrium within the observation window. While this suggests non-equilibrium dynamics, the resulting free energy landscape is presented as a qualitative visualization of conformational trends rather than a representation of fully converged thermodynamics. This helps compare sampling behavior between wild-type and variant forms and should be interpreted accordingly.

## 4 Discussion

### 4.1 Genetic Characterization of LDLR Variants Identified in FH Families

FH is a monogenic disorder characterized by the loss of low-density lipoprotein receptor (LDLR) function in either homozygous or heterozygous forms. This study identified two such variants: a frameshift variant(c.2416dup) in family A and a nonsense variant (c.103 C>T) in family B. Both variants disrupt the LDLR protein and were found only in family members with significantly severe high cholesterol levels. In the two families, the index patients with a DLCN score indicating probable FH exhibited a significant difference in LDL-C levels based on biochemical analysis. Since genetic testing is the standard for diagnosing FH, these findings strongly suggest that c.2416dup and c.103 C>T variants directly contribute to the development of the disease. LDLR is a transmembrane glycoprotein with a critical role in cholesterol homeostasis. The mature LDLR protein comprises approximately 860 amino acids and consists of well-defined functional domains: ligand-binding, epidermal growth factor (EGF)-like precursor homology, oligosaccharide-binding, transmembrane, and cytoplasmic domains [26]. Class 1 LDLR variants encompassing nonsense mutations, splice site alterations, exonic deletions/duplications, and frameshift mutations can lead to null alleles [26], [27], [28]. The specific impact on LDLR function depends on the affected protein domain and the resulting disruption of its critical activities [29].

The c.2416dup variant, which affects the membrane-anchoring and cytoplasmic tail domains, can lead to nonsense-mediated mRNA decay and impaired LDLR function [30], [31]. The transmembrane domain (encoded by exon 16 and the 5′-end of exon 17) anchors LDLR to the cell membrane. The cytoplasmic domain (encoded by the 3′ end of exon 17 and the 5′ end of exon 18) is the most conserved region of the protein and regulates LDLR trafficking to the cell surface and restrains the receptor in the coated pits [30], [32], [33]. Only 5.9 % of allelic variants have been reported within this domain, which is 86 % identical among mammals [34]. Changes in the cytoplasmic domain length can decrease LDLRs on the cell surface. An in vitro study suggested that the cytoplasmic domain needs at least 30 residues for proper folding and the exit of LDLR from the endoplasmic reticulum, potentially controlling phagocytosis [35], [36]. The cytoplasmic domain is critical for LDLR function in cholesterol metabolism. Alteration in the C-terminal cytoplasmic tail and disrupts the NPXY internalization motif, previous studies have shown that such tail-truncated LDLR mutants retain normal LDL binding but fail to cluster in coated pits, leading to impaired endocytosis and complete loss of LDL-C degradation [37], [38].

The c.103 C>T variant is in the ligand-binding domain of the LDLR gene, which is encoded by exons 2–6 and consists of approximately 300 amino acid residues. This domain plays a crucial role in LDL recognition and clearance, as it contains the binding sites for apolipoproteins B and E, essential for LDL uptake [39], [40]. Structurally, the ligand-binding domain is composed of seven highly conserved tandem repeats (LA1-LA7), each approximately 40 residues long and stabilized by six disulfide bonds. Functional studies indicate that LA3-LA7 are critical for apoB-100 binding, while LA4-LA5 are specifically involved in LDL release within endosomes [41]. The disruption caused by nonsense variants, such as c.103 C>T, further reinforces the importance of *LDLR* structural integrity in cholesterol homeostasis. A recent study on a similar exon 2 nonsense variant (p.Gln33Ter) demonstrated a > 95 % reduction in LDLR expression by Western blot and a complete absence of surface LDLR by flow cytometry in transfected cells, supporting the pathogenicity of variants affecting this site [42].

### 4.2 Genotype-Phenotype Correlation

In family A, individuals carrying the c. 2416dup variant had markedly higher levels (352 mg/dL) compared to those without the variant (138 mg/dL). The proband's mother (II-2) did not carry the variant, this suggests paternal inheritance of the disease variants. Furthermore, the elevated LDL-C associated with c.2416dup potentially explains the young age at which the deceased father (II-3) and two siblings (III-4 and III-16) experienced heart attacks (all under 40 years old). Previous studies have linked the c.2416dup variant to severe clinical manifestations and an increased risk of coronary heart disease, further reinforcing this association [43], [44], [45], [46]. Research suggests that the specific genetic makeup of FH patients influences the severity of symptoms. Patients with homozygous FH (HoFH) experience cardiovascular complications at a very young age, often during childhood or adolescence [17], [47], [48]. Conversely, individuals with heterozygous FH, like those in Family A with the c.2416dup variant, may not exhibit these symptoms until later in life.

The c.2416dup variant was first reported in FH patients from Swedish and American families in 1998 and 1999, respectively [49], [50]. Interestingly, the variant (c.2416dup) has been found in fifty-five individuals from diverse populations, including Polish, Indian, British, Hungarian, Brazilian, Swedish, Turkish, Chinese, Pakistani, Japanese, American, Russian, and Norwegian. Conversely, the c.103 C>T variant was documented only in Italian FH patients [51], [52]. However, these findings have not been reported in any Saudi patient [16], [17], [53], [54], [55], [56], [57], [58] and our study is the first to report this variant in Saudi FH patients. Additionally, this variant has not been reported in Arab patients [59], except in a homozygous state (c.2416dup) in two individuals from the Middle East, as documented in an Iranian study [30], [60].

Individuals in family B carrying the c.103 C>T variant exhibited significantly elevated LDL-C levels (286 mg/dL) compared to those without the variant (117 mg/dL). This elevation in LDL-C may be associated with the presence of arcus senilis, a recognized clinical sign of FH [61], observed in the father (I-2) of Family B. Understanding the molecular consequences of *LDLR* variants provides valuable insights into the pathogenesis of FH and informs targeted therapeutic strategies [62].

### 4.3 Structural and Functional Consequences: In Silico Insights

The RNAfold analysis conducted in our study unveils substantial structural differences among LDLR gene variants, notably in terms of MFE and ensemble diversity. Higher MFE observed in mutants indicates less stable RNA structures, potentially influencing gene expression and RNA splicing efficiency. These alterations in RNA stability might impact cellular functions [63], [64]. The study of Ding et al. noted the magnitudes of MFE differences in the mutant RNA structure [65], [66]. These studies highlight that even minor variations in RNA folding energy can affect mRNA stability, splicing efficiency, and ultimately protein expression. Therefore, the differences we observed are consistent with the expected impact of such variants on RNA structure and function. Increased ensemble diversity in mutants’ hints at heightened structural variability, potentially resulting in varied functional outcomes or interactions within cells.

The docking simulation revealed a distinct set of hydrogen bond contacts (Asn825–Lys87, Gly829–Leu107, Asp853–Ser81, Glu856–Thr105) that localize in the cytoplasmic tail of the LDLR, in immediate proximity to the transmembrane helix. This sequence encompasses the NPxY internalization motif (residues 807–810), the prototypic docking site for the phosphotyrosine-binding (PTB) domain of the LDLRAP1 [67], [68]. Stabilization interactions we observed with residues such as Asp853 and Glu856 suggest that contacts extend beyond the core NPxY, consistent with structural work revealing that LDLRAP1 recognizes an expanded binding patch (I−7xF−5xNPxY⁰QK⁺²) of the LDLR over the four-residue motif in a vacuum. Such an expanded interface offers a combination of electrostatic complementarity plus backbone stabilization, explaining the relatively modest docking energy (−218.6 kJ/mol) observed in our simulation. Interestingly, the c.2416dup frameshift eliminates the whole region, effacing not only the string of the NPxY sequence but additionally the flanking acidic residues responsible for the stabilization of the LDLR–LDLRAP1 complex. This frameshift mutation resulting in truncation of this region is likely to disrupt these essential non-covalent interactions, compromising the structural integrity of the LDLR-LDLRAP1 complex. This disruption may impair receptor internalization, recycling, and degradation, thereby contributing to dysfunctional cholesterol homeostasis. These findings are consistent with previous studies highlighting the essential role of the intracellular domain in LDLR trafficking [69], [70]. Notably, while truncated LDLR proteins may undergo rapid degradation due to misfolding, some may remain stable but escape LDLRAP1-mediated endocytosis, resulting in elevated cell-surface LDLR levels with impaired internalization [71].

Molecular-dynamics simulations, furthermore, showed that the c.2416dup frameshift (p.Val806GlyfsTer11) deletes the H18 transmembrane/juxtacytosolic segment and the bulk of the tail, eliminating the NPXY region and matching distal contact residues, inducing increased near-truncation flexibility (RMSD, RMSF), and destabilizing the internalization interface. In MD alteration in secondary structure, particularly the reduction in beta sheet content, underscores the variant's disruptive impact on the receptor's conformation. The disruption of beta-sheet content in the 3′ region of the LDLR protein can have significant biological implications. Beta sheets are crucial for the structural integrity and stability of proteins. The observed reduction in beta sheet content in the mutant protein may lead to a less stable and more flexible structure. This alteration could impact on the protein's ability to maintain its proper conformation and function, potentially affecting its interactions with other molecules and its overall role in cellular processes.

PCA and free energy landscapes further elucidated the distinct conformational behaviors between the wild-type and mutant forms, with the mutant exhibiting altered dynamic behavior and thermodynamic properties. These findings suggest that the truncation variant fundamentally alters LDLR's stability and function, with implications for its biological activity and the pathological basis of related diseases. Our RNA and protein structural analyses shed light on *LDLR* gene variants, emphasizing their potential significance in cholesterol regulation and associated metabolic pathways. Given LDLR's pivotal role in cholesterol homeostasis, observed structural changes due to variants might contribute to dyslipidemia and related cardiovascular conditions. These findings underscore the intricate relationship between genetic variations, molecular structure, and disease mechanisms, emphasizing the importance of detailed molecular analyses in understanding genetic disorders.

Recent bioinformatics approaches emphasize the role of molecular dynamics simulations and protein docking models in assessing the structural and functional impacts of *LDLR* variants associated with FH. These techniques provide valuable insights into how variants alter LDLR conformation and binding interactions, helping to predict their pathogenicity, though experimental validation remains crucial for confirming computational findings [72], [73]. A Study by Fisher et al., revealed the detailed structural features of LDLR and its interaction with other lipoproteins, which provides new insights into LDLR-mediated endocytosis using cryo-electron microscopy [74]. The study also identified the key amino acid residues involved in the LDLR-lipoprotein interaction, which could be targeted for therapeutic interventions in cardiovascular diseases. Awan et al., used a combination of experimental and computational methods to investigate the structural and functional implications of LDLR variants in familial hypercholesterolemia [17]. They demonstrated that some LDLR variants exhibited structural instability and impaired LDL binding leading to the accumulation of cholesterol in the blood vessels and increasing the risk of cardiovascular diseases.

We wish to acknowledge that due to the challenging and time-consuming nature of functional characterization experiments in cell lines or animal models, we were unable to functionally characterize the *LDLR* c.2416dup and c.103 C>T variants. Using established computational tools that have demonstrated efficacy in predicting the functional impact of variants on protein structure and function experiments in cell lines or animal models [75]. These computational methods provided valuable insights into the potential consequences of the identified variants on LDLR function, complementing the clinical and genetic data presented in this study.

### 4.4 Limitations and Future Perspectives

This study sincerely acknowledges a few limitations. It is based on two families and lacks in vivo functional validation, with findings based primarily on computational predictions. Furthermore, functional assays such as LDLR expression, Western blotting, or LDL-C uptake in patient-derived cells were not feasible due to logistical constraints and lack of viable samples, representing a key limitation in confirming the predicted molecular effects. Also, our computational MD analysis is that membrane-embedded simulations were not performed. Although LDLR is a membrane protein, the simulations were conducted in aqueous conditions to examine global conformational dynamics. In future studies, we aim to explore more complex membrane-bound dynamics, including LDLR-LDLRAP1 interactions within a lipid bilayer environment. Additionally, Future studies with larger cohorts and experimental validation are needed to confirm these results and expand our understanding of genotype-phenotype correlations in FH.

## 5 Conclusion

In conclusion, this study identifies two rare pathogenic LDLR variants, c.2416dup and c.103 C>T, in an extended Saudi Arabian family. These findings offer potential benefits such as early identification of FH cases, lipid-lowering therapy planning, and genetic counselling. The molecular diagnosis of this c.2416dup and c.103 C>T variants could also help design a custom FH SNP panel for the Saudi population in the context of precision medicine. The study demonstrates the integration of bioinformatics methods with sequencing data to characterize familial hypercholesterolemia causative proteins. Nevertheless, further experimental validation is necessary to confirm computational predictions and investigate the structural and functional implications of LDLR genetic variations.

## Ethical approval

This study was approved by the ethics committee of King Abdulaziz University, Saudi Arabia. Informed consent was obtained from each tested individual prior to genetic testing.

## Disclosure of Funding

This publication is based upon work supported by the 10.13039/501100004070Khalifa University (KU) and 10.13039/501100004054King Abdulaziz University (KAU) Join Research Program No. KAUKUJRP-1M-2021. The authors, therefore, acknowledge the Deanship of Scientific Research (10.13039/501100000956DSR), KAU for technical and financial support.

## Author Agreement Statement

We the undersigned declare that this manuscript is original, has not been published before and is not currently being considered for publication elsewhere.

We confirm that the manuscript has been read and approved by all named authors and that there are no other persons who satisfied the criteria for authorship but are not listed.

We further confirm that the order of authors listed in the manuscript has been approved by all of us.

We understand that the Corresponding Author is the sole contact for the Editorial process.

## CRediT authorship contribution statement

**Abdulrahman Mujalli:** Writing – original draft, Methodology. **Safa Bayashut:** Resources, Project administration. **Alaa Y Edrees:** Investigation. **Awan Dr Zuhier:** Writing – original draft, Supervision, Investigation. **Babajan Banaganapalli:** Writing – original draft, Software, Methodology. **Sherif Edris:** Supervision, Formal analysis. **Eman Alefishat:** Funding acquisition. **Hadiah Bassam Al Mahdi:** Writing – original draft, Methodology. **Ramu Elango:** Writing – review & editing. **Noor Ahmad Shaik:** Writing – review & editing, Writing – original draft, Funding acquisition. **Hussam Daghistani:** Writing – original draft, Data curation. **Yousef Almoghrabi:** Writing – review & editing, Investigation. **Rawabi Zahed:** Writing – review & editing, Methodology. **Hanan Abdelhalim ElSokary:** Project administration, Data curation.

## Declaration of Competing Interest

The authors declare that they have no known competing financial interests or personal relationships that could have appeared to influence the work reported in this paper.

## Data Availability

All datasets analyzed for this study are included in the article.

## References

[1] Baruah D.K. (2021). Familial hypercholesterolemia. Heart Views.

[2] Chacón-Camacho O.F., Pozo-Molina G., Méndez-Catalá C.F., Reyes-Reali J., Méndez-Cruz R., Zenteno J.C. (2022). Familial hypercholesterolemia: update and review. Endocr Metab Immune Disord Drug Targets.

[3] Lee S.-H. (2017). Update on familial hypercholesterolemia: diagnosis, cardiovascular risk, and novel therapeutics. Endocrinol Metab (Seoul).

[4] Marziliano N., Medoro A., Folzani S., Intrieri M., Reverberi C. (2022). Molecular genetics for familial hypercholesterolemia. Rev Cardiovasc Med.

[5] Alhabib K.F., Al-Rasadi K., Almigbal T.H., Batais M.A., Al-Zakwani I., Al-Allaf F.A. (2021). Familial hypercholesterolemia in the Arabian Gulf region: clinical results of the gulf FH registry. PLoS One.

[6] Wilemon K.A., Patel J., Aguilar-Salinas C., Ahmed C.D., Alkhnifsawi M., Almahmeed W. (2020). Reducing the clinical and public health burden of familial hypercholesterolemia: a global call to action. JAMA Cardiol.

[7] Tokgozoglu L., Kayikcioglu M. (2021). Familial hypercholesterolemia: global burden and approaches. Curr Cardiol Rep.

[8] Al-Rasadi K., Alhabib K.F., Al-Allaf F., Al-Waili K., Al-Zakwani I., AlSarraf A. (2020). The gulf familial hypercholesterolemia registry (Gulf FH): design, rationale and preliminary results. Curr Vasc Pharm.

[9] Bamimore M.A., Zaid A., Banerjee Y., Al-Sarraf A., Abifadel M., Seidah N.G. (2015). Familial hypercholesterolemia mutations in the middle eastern and north African region: a need for a national registry. J Clin Lipido.

[10] Stajkovska A., Mehandziska S., Stavrevska M., Jakovleva K., Nikchevska N., Mitrev Z. (2018). Trio clinical exome sequencing in a patient with multicentric carpotarsal osteolysis syndrome: first case report in the balkans. Front Genet.

[11] Shaik N.A., Al-Shehri N., Athar M., Awan A., Khalili M., Al Mahadi H.B. (2023). Protein structural insights into a rare PCSK9 gain-of-function variant (R496W) causing familial hypercholesterolemia in a Saudi family: whole exome sequencing and computational analysis. Front Physiol.

[12] Ulintz P.J., Wu W., Gates C.M. (2019). Bioinformatics analysis of whole exome sequencing data. Methods Mol Biol.

[13] Chang P.L. (2005). Clinical bioinformatics. Chang Gung Med J.

[14] Thirumal Kumar D., Udhaya Kumar S., Jain N., Sowmya B., Balsekar K., Siva R. (2022). Computational structural assessment of BReast CAncer type 1 susceptibility protein (BRCA1) and BRCA1-Associated ring domain protein 1 (BARD1) mutations on the protein-protein interface. Adv Protein Chem Struct Biol.

[15] Thirumal Kumar D., Iyer S., Christy J.P., Siva R., Tayubi I.A., George Priya Doss C. (2019). A comparative computational approach toward pharmacological chaperones (NN-DNJ and ambroxol) on N370S and L444P mutations causing Gaucher’s disease. Adv Protein Chem Struct Biol.

[16] Awan Z., Batran A., Al-Allaf F.A., Alharbi R.S., Hegazy G.A., Jamalalail B. (2023). Identification and functional characterization of two rare LDLR stop gain variants (p.C231* and p.R744*) in Saudi familial hypercholesterolemia patients. Panminerva Med.

[17] Awan Z.A., Rashidi O.M., Al-Shehri B.A., Jamil K., Elango R., Al-Aama J.Y. (2021). Saudi familial hypercholesterolemia patients with rare LDLR stop gain variant showed variable clinical phenotype and resistance to multiple drug regimen. Front Med (Lausanne).

[18] Austin M.A., Hutter C.M., Zimmern R.L., Humphries S.E. (2004). Genetic causes of monogenic heterozygous familial hypercholesterolemia: a HuGE prevalence review. Am J Epidemiol.

[19] Defesche J.C., Gidding S.S., Harada-Shiba M., Hegele R.A., Santos R.D., Wierzbicki A.S. (2017). Familial hypercholesterolaemia. Nat Rev Dis Prim.

[20] Li H., Durbin R. (2010). Fast and accurate long-read alignment with Burrows-Wheeler transform. Bioinformatics.

[21] Richards S., Aziz N., Bale S., Bick D., Das S., Gastier-Foster J. (2015). Standards and guidelines for the interpretation of sequence variants: a joint consensus recommendation of the American college of medical genetics and genomics and the association for molecular pathology. Genet Med.

[22] Gruber A.R., Lorenz R., Bernhart S.H., Neuböck R., Hofacker I.L. (2008). The Vienna RNA websuite. Nucleic Acids Res.

[23] Thompson B., Petrić Howe N. (2024). Alphafold 3.0: the AI protein predictor gets an upgrade. Nature.

[24] Jensen M.Ø., Borhani D.W., Lindorff-Larsen K., Maragakis P., Jogini V., Eastwood M.P. (2010). Principles of conduction and hydrophobic gating in K+ channels. Proc Natl Acad Sci USA.

[25] Mark P., Nilsson L. (2001). Structure and dynamics of the TIP3P, SPC, and SPC/E water models at 298 k. J Phys Chem A.

[26] Vrablik M., Tichý L., Freiberger T., Blaha V., Satny M., Hubacek J.A. (2020). Genetics of familial hypercholesterolemia: new insights. Front Genet.

[27] Abifadel M., Boileau C. (2023). Genetic and molecular architecture of familial hypercholesterolemia. J Intern Med.

[28] Benito-Vicente A., Uribe K.B., Jebari S., Galicia-Garcia U., Ostolaza H., Martin C. (2018). Validation of LDLr activity as a tool to improve genetic diagnosis of familial hypercholesterolemia: a retrospective on functional characterization of LDLr variants. Int J Mol Sci.

[29] Turkyilmaz A., Kurnaz E., Alavanda C., Yarali O., Kartal Baykan E., Yavuz D. (2021). The spectrum of Low-Density lipoprotein receptor mutations in a large turkish cohort of patients with familial hypercholesterolemia. Metab Syndr Relat Disord.

[30] Fairoozy R.H., Futema M., Vakili R., Abbaszadegan M.R., Hosseini S., Aminzadeh M. (2017). The genetic spectrum of familial hypercholesterolemia (FH) in the Iranian population. Sci Rep.

[31] Dvir H., Shah M., Girardi E., Guo L., Farquhar M.G., Zajonc D.M. (2012). Atomic structure of the autosomal recessive hypercholesterolemia phosphotyrosine-binding domain in complex with the LDL-receptor tail. Proc Natl Acad Sci USA.

[32] Corbin I.R., Li H., Chen J., Lund-Katz S., Zhou R., Glickson J.D. (2006). Low-density lipoprotein nanoparticles as magnetic resonance imaging contrast agents. Neoplasia.

[33] Oommen D., Kizhakkedath P., Jawabri A.A., Varghese D.S., Ali B.R. (2020). Proteostasis regulation in the endoplasmic reticulum: an emerging theme in the molecular pathology and therapeutic management of familial hypercholesterolemia. Front Genet.

[34] De Castro-Orós I., Pocoví M., Civeira F. (2010). The genetic basis of familial hypercholesterolemia: inheritance, linkage, and mutations. Appl Clin Genet.

[35] Patel M., Morrow J., Maxfield F.R., Strickland D.K., Greenberg S., Tabas I. (2003). The cytoplasmic domain of the low density lipoprotein (LDL) receptor-related protein, but not that of the LDL receptor, triggers phagocytosis. J Biol Chem.

[36] Singh S., Singh M., Dayal D., Bhatia P., Negi S., V Attri S. (2021). Characterisation of LDL receptor gene mutations in a north Indian cohort of children with homozygous familial hypercholesterolaemia. Pedia Endocrinol Diabetes Metab.

[37] Davis C.G., Lehrman M.A., Russell D.W., Anderson R.G., Brown M.S., Goldstein J.L. (1986). The J.D. Mutation in familial hypercholesterolemia: amino acid substitution in cytoplasmic domain impedes internalization of LDL receptors. Cell.

[38] Lehrman M.A., Goldstein J.L., Brown M.S., Russell D.W., Schneider W.J. (1985). Internalization-defective LDL receptors produced by genes with nonsense and frameshift mutations that truncate the cytoplasmic domain. Cell.

[39] Südhof T.C., Goldstein J.L., Brown M.S., Russell D.W. (1985). The LDL receptor gene: a mosaic of exons shared with different proteins. Science.

[40] Varret M., Rabès J.-P., Varret M., Rabès J.-P. (2012). Mutations in Human Genetic Disease.

[41] Jiang L., Benito-Vicente A., Tang L., Etxebarria A., Cui W., Uribe K.B. (2017). Analysis of LDLR variants from homozygous FH patients carrying multiple mutations in the LDLR gene. Atherosclerosis.

[42] Wang K., Hu T., Tai M., Shen Y., Lin S., Guo Y. (2024). Pathogenicity of the LDLR c.97C>T (p.Gln33Ter) mutation in familial hypercholesterolemia. Mol Genet Genom Med.

[43] Wald D.S., Bangash F.A., Bestwick J.P. (2015). Prevalence of DNA-confirmed familial hypercholesterolaemia in young patients with myocardial infarction. Eur J Intern Med.

[44] Setia N., Saxena R., Sawhney J.P.S., Verma I.C. (2018). Familial hypercholesterolemia: cascade screening in children and relatives of the affected. Indian J Pedia.

[45] Miyake Y., Yamamura T., Sakai N., Miyata T., Kokubo Y., Yamamoto A. (2009). Update of Japanese common LDLR gene mutations and their phenotypes: mild type mutation L547V might predominate in the Japanese population. Atherosclerosis.

[46] Setia N., Saxena R., Arora A., Verma I.C. (2016). Spectrum of mutations in homozygous familial hypercholesterolemia in India, with four novel mutations. Atherosclerosis.

[47] Goldstein J.L., Brown M.S. (2001). Molecular Medicine. The cholesterol quartet. Science.

[48] Cuchel M., Bruckert E., Ginsberg H.N., Raal F.J., Santos R.D., Hegele R.A. (2014). Homozygous familial hypercholesterolaemia: new insights and guidance for clinicians to improve detection and clinical management. A position paper from the consensus panel on familial hypercholesterolaemia of the european atherosclerosis society. Eur Heart J.

[49] Ekström U., Abrahamson M., Wallmark A., Florén C.H., Nilsson-Ehle P. (1998). Mutations in the low-density lipoprotein receptor gene in Swedish familial hypercholesterolaemia patients: clinical expression and treatment response. Eur J Clin Invest.

[50] Nobe Y., Emi M., Katsumata H., Nakajima T., Hirayama T., Wu L.L. (1999). Familial hypercholesterolemia in utah kindred with novel 2412-6 ins g mutations in exon 17 of the LDL receptor gene. Jpn Heart J.

[51] Bertolini S., Cassanelli S., Garuti R., Ghisellini M., Simone M.L., Rolleri M. (1999). Analysis of LDL receptor gene mutations in Italian patients with homozygous familial hypercholesterolemia. Arterioscler Thromb Vasc Biol.

[52] Bertolini S., Cantafora A., Averna M., Cortese C., Motti C., Martini S. (2000). Clinical expression of familial hypercholesterolemia in clusters of mutations of the LDL receptor gene that cause a receptor-defective or receptor-negative phenotype. Arterioscler Thromb Vasc Biol.

[53] Al-Allaf F.A., Alashwal A., Abduljaleel Z., Taher M.M., Bouazzaoui A., Abalkhail H. (2017). Compound heterozygous LDLR variant in severely affected familial hypercholesterolemia patient. Acta Biochim Pol.

[54] Al-Allaf F., Athar M., Alashwal A., Abduljaleel Z., Mohiuddin T., Bouazzaoui A. (2017). 1. Founder mutation identified in the LDLR gene causing familial hypercholesterolemia associated with increased risk of coronary heart disease. J Saudi Heart Assoc.

[55] Al-Allaf F.A., Alashwal A., Abduljaleel Z., Taher M.M., Siddiqui S.S., Bouazzaoui A. (2016). Identification of a recurrent frameshift mutation at the LDLR exon 14 (c.2027delG, p.(G676Afs*33)) causing familial hypercholesterolemia in Saudi Arab homozygous children. Genomics.

[56] Al-Allaf F.A., Athar M., Abduljaleel Z., Bouazzaoui A., Taher M.M., Own R. (2014). Identification of a novel nonsense variant c.1332dup, p.(D445*) in the LDLR gene that causes familial hypercholesterolemia. Hum Genome Var.

[57] Al-Allaf F.A., Athar M., Abduljaleel Z., Taher M.M., Khan W., Ba-Hammam F.A. (2015). Next generation sequencing to identify novel genetic variants causative of autosomal dominant familial hypercholesterolemia associated with increased risk of coronary heart disease. Gene.

[58] Alnouri F., Al-Allaf F.A., Athar M., Abduljaleel Z., Alabdullah M., Alammari D. (2020). Xanthomas can be misdiagnosed and mistreated in homozygous familial hypercholesterolemia patients: a call for increased awareness among dermatologists and health care practitioners. Glob Heart.

[59] Awan Z.A., Bondagji N.S., Bamimore M.A. (2019). Recently reported familial hypercholesterolemia-related mutations from cases in the Middle East and north Africa region. Curr Opin Lipido.

[60] Mahdieh N., Heshmatzad K., Rabbani B. (2020). A systematic review of LDLR, PCSK9, and APOB variants in Asia. Atherosclerosis.

[61] Rallidis L.S., Iordanidis D., Iliodromitis E. (2020). The value of physical signs in identifying patients with familial hypercholesterolemia in the era of genetic testing. J Cardiol.

[62] Tveten K., Holla Ø.L., Cameron J., Strøm T.B., Berge K.E., Laerdahl J.K. (2012). Interaction between the ligand-binding domain of the LDL receptor and the C-terminal domain of PCSK9 is required for PCSK9 to remain bound to the LDL receptor during endosomal acidification. Hum Mol Genet.

[63] Singh A.B., Li H., Kan C.F.K., Dong B., Nicolls M.R., Liu J. (2014). The critical role of mRNA destabilizing protein heterogeneous nuclear ribonucleoprotein d in 3’ untranslated region-mediated decay of low-density lipoprotein receptor mRNA in liver tissue. Arterioscler Thromb Vasc Biol.

[64] Wilkinson E., Cui Y.-H., He Y.-Y. (2022). Roles of RNA modifications in diverse cellular functions. Front Cell Dev Biol.

[65] Ding Y., Chan C.Y., Lawrence C.E. (2004). Sfold web server for statistical folding and rational design of nucleic acids. Nucleic Acids Res.

[66] Wan Y., Kertesz M., Spitale R.C., Segal E., Chang H.Y. (2011). Understanding the transcriptome through RNA structure. Nat Rev Genet.

[67] Chen W.J., Goldstein J.L., Brown M.S. (1990). NPXY, a sequence often found in cytoplasmic tails, is required for coated pit-mediated internalization of the low density lipoprotein receptor. J Biol Chem.

[68] Bansal A., Gierasch L.M. (1991). The NPXY internalization signal of the LDL receptor adopts a reverse-turn conformation. Cell.

[69] Mikaeeli S., Ben Djoudi Ouadda A., Evagelidis A., Essalmani R., Ramos O.H.P., Fruchart-Gaillard C. (2024). Insights into PCSK9-LDLR regulation and trafficking via the differential functions of MHC-I proteins HFE and HLA-C. Cells.

[70] Aldworth H., Hooper N.M. (2024). Post-translational regulation of the low-density lipoprotein receptor provides new targets for cholesterol regulation. Biochem Soc Trans.

[71] Xia X.-D., Peng Z.-S., Gu H.-M., Wang M., Wang G.-Q., Zhang D.-W. (2021). Regulation of PCSK9 expression and function: mechanisms and therapeutic implications. Front Cardiovasc Med.

[72] Larrea-Sebal A., Jebari-Benslaiman S., Galicia-Garcia U., Jose-Urteaga A.S., Uribe K.B., Benito-Vicente A. (2023). Predictive modeling and structure analysis of genetic variants in familial hypercholesterolemia: implications for diagnosis and protein interaction studies. Curr Atheroscler Rep.

[73] Klee E.W., Zimmermann M.T. (2019). Molecular modeling of LDLR aids interpretation of genomic variants. J Mol Med (Berl).

[74] Fisher C., Beglova N., Blacklow S.C. (2006). Structure of an LDLR-RAP complex reveals a general mode for ligand recognition by lipoprotein receptors. Mol Cell.

[75] George Priya Doss C., Chakraborty C., Narayan V., Thirumal Kumar D. (2014). Computational approaches and resources in single amino acid substitutions analysis toward clinical research. Adv Protein Chem Struct Biol.

[76] Sturm A.C., Truty R., Callis T.E., Aguilar S., Esplin E.D., Garcia S. (2021). Limited-Variant screening vs comprehensive genetic testing for familial hypercholesterolemia diagnosis. JAMA Cardiol.

[77] Jannes C.E., Santos R.D., de Souza Silva P.R., Turolla L., Gagliardi A.C.M., Marsiglia J.D.C. (2015). Familial hypercholesterolemia in Brazil: cascade screening program, clinical and genetic aspects. Atherosclerosis.

[78] Xiang R., Fan L.-L., Lin M.-J., Li J.-J., Shi X.-Y., Jin J.-Y. (2017). The genetic spectrum of familial hypercholesterolemia in the central south region of China. Atherosclerosis.

[79] Fan L., Lin M., Chen Y., Huang H., Peng D., Xia K. (2015). Novel mutations of low-density lipoprotein receptor gene in China patients with familial hypercholesterolemia. Appl Biochem Biotechnol.

[80] Kuhrová V., Francová H., Zapletalová P., Freiberger T., Fajkusová L., Hrabincová E. (2001). Spectrum of low density lipoprotein receptor mutations in Czech hypercholesterolemic patients. Hum Mutat.

[81] Madar L., Juhász L., Szűcs Z., Kerkovits L., Harangi M., Balogh I. (2022). Establishing the mutational spectrum of Hungarian patients with familial hypercholesterolemia. Genes (Basel).

[82] Tada H., Hori M., Nomura A., Hosomichi K., Nohara A., Kawashiri M.-A. (2020). A catalog of the pathogenic mutations of LDL receptor gene in Japanese familial hypercholesterolemia. J Clin Lipido.

[83] Hori M., Ohta N., Takahashi A., Masuda H., Isoda R., Yamamoto S. (2019). Impact of LDLR and PCSK9 pathogenic variants in Japanese heterozygous familial hypercholesterolemia patients. Atherosclerosis.

[84] Leren T.P., Bogsrud M.P. (2021). Molecular genetic testing for autosomal dominant hypercholesterolemia in 29,449 Norwegian index patients and 14,230 relatives during the years 1993-2020. Atherosclerosis.

[85] Ajmal M., Ahmed W., Sadeque A., Ali S.H.B., Bokhari S.H., Ahmed N. (2010). Identification of a recurrent insertion mutation in the LDLR gene in a Pakistani family with autosomal dominant hypercholesterolemia. Mol Biol Rep.

[86] Rutkowska, Sałacińska L., Salachna K., Matusik D., Pinkier P., Kępczyński I. (2022). Identification of new genetic determinants in pediatric patients with familial hypercholesterolemia using a custom NGS panel. Genes (Basel).

[87] Meshkov A., Ershova A., Kiseleva A., Zotova E., Sotnikova E., Petukhova A. (2021). The LDLR, APOB, and PCSK9 variants of index patients with familial hypercholesterolemia in russia. Genes (Basel).

[88] Semenova A.E., Sergienko I.V., García-Giustiniani D., Monserrat L., Popova A.B., Nozadze D.N. (2020). Verification of underlying genetic cause in a cohort of Russian patients with familial hypercholesterolemia using targeted next generation sequencing. J Cardiovasc Dev Dis.

[89] Benedek P., Jiao H., Duvefelt K., Skoog T., Linde M., Kiviluoma P. (2021). Founder effects facilitate the use of a genotyping-based approach to molecular diagnosis in Swedish patients with familial hypercholesterolaemia. J Intern Med.

