# Supplementary material for: Pathogenic LDLR Variants (c.103 C>T and c.2416dup) in ligand-binding and cytosolic domains in Saudi familial hypercholesterolemia: Molecular characterization and computational insights
Source: Comput Struct Biotechnol J. 2025 Aug 25;27:3770–84. doi: 10.1016/j.csbj.2025.08.029 (PMC12447921; doi:10.1016/j.csbj.2025.08.029)
Supplement: Supplementary file 1 — Supplementary material [file mmc1.docx]

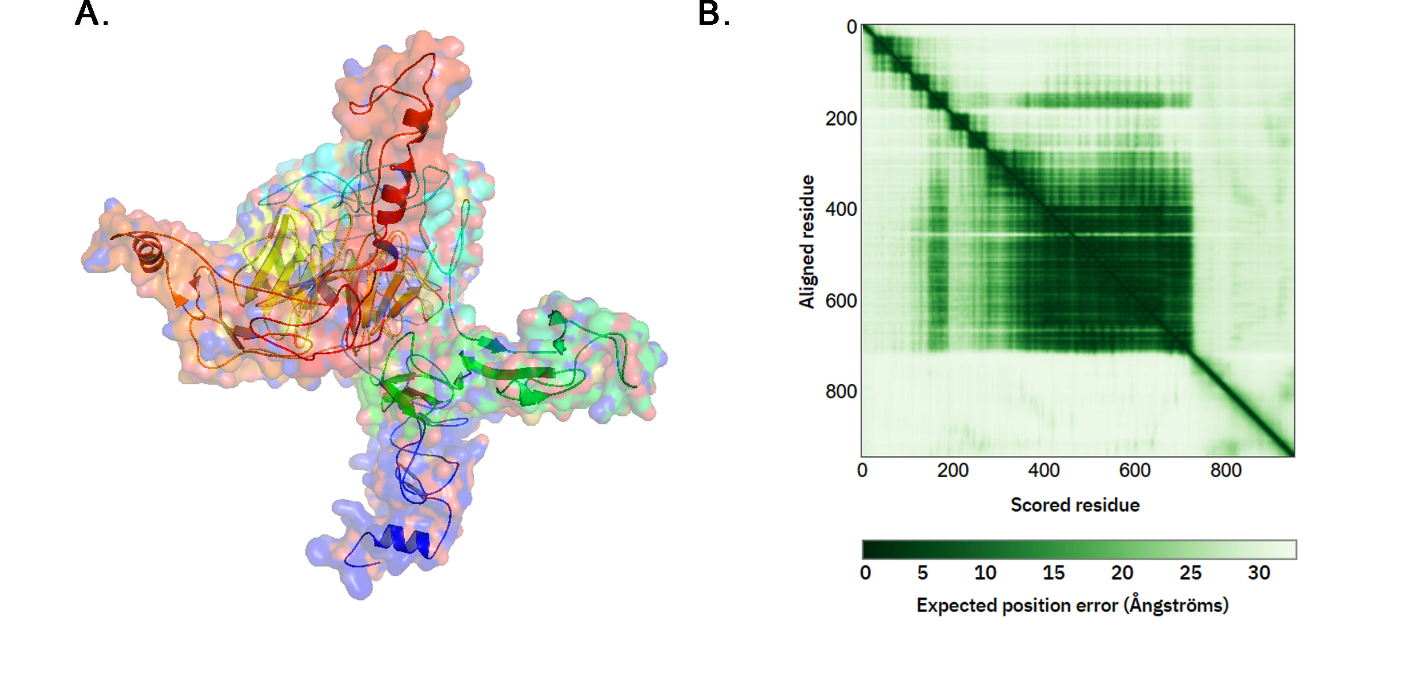


**S1 Fig :** **AlphaFold Predictions for LDLR Protein:** A. Alphafold generated full length LDLR protein structure B. Residue-residue alignment confidence (PAE) plot of LDLR predicted model. At residue position y, the color represents AlphaFold's anticipated error in predicting residue position x when the true and predicted structures are aligned.

**
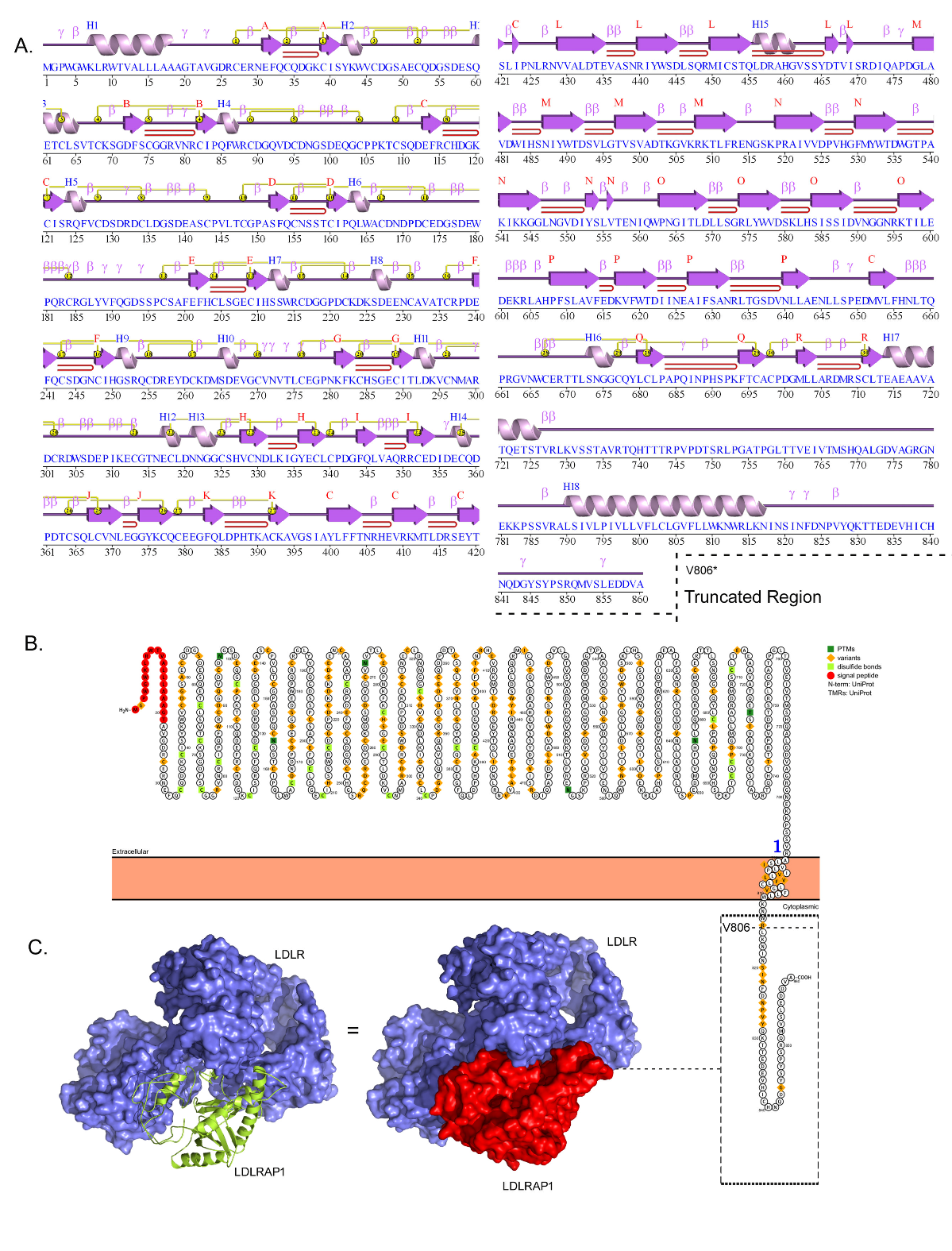
**

**S2 Fig : Structural Analysis of LDLR Protein and its Interaction with** **LDLRAP1:** A. PDBSUM shows the secondary structural components of the AlphaFold-generated LDLR protein B. The Protter tool predicts the transmembrane topology of LDLR protein C. The circle plots in docking illustrate the interaction of the LDLR truncated peptide with LDLRAP1.


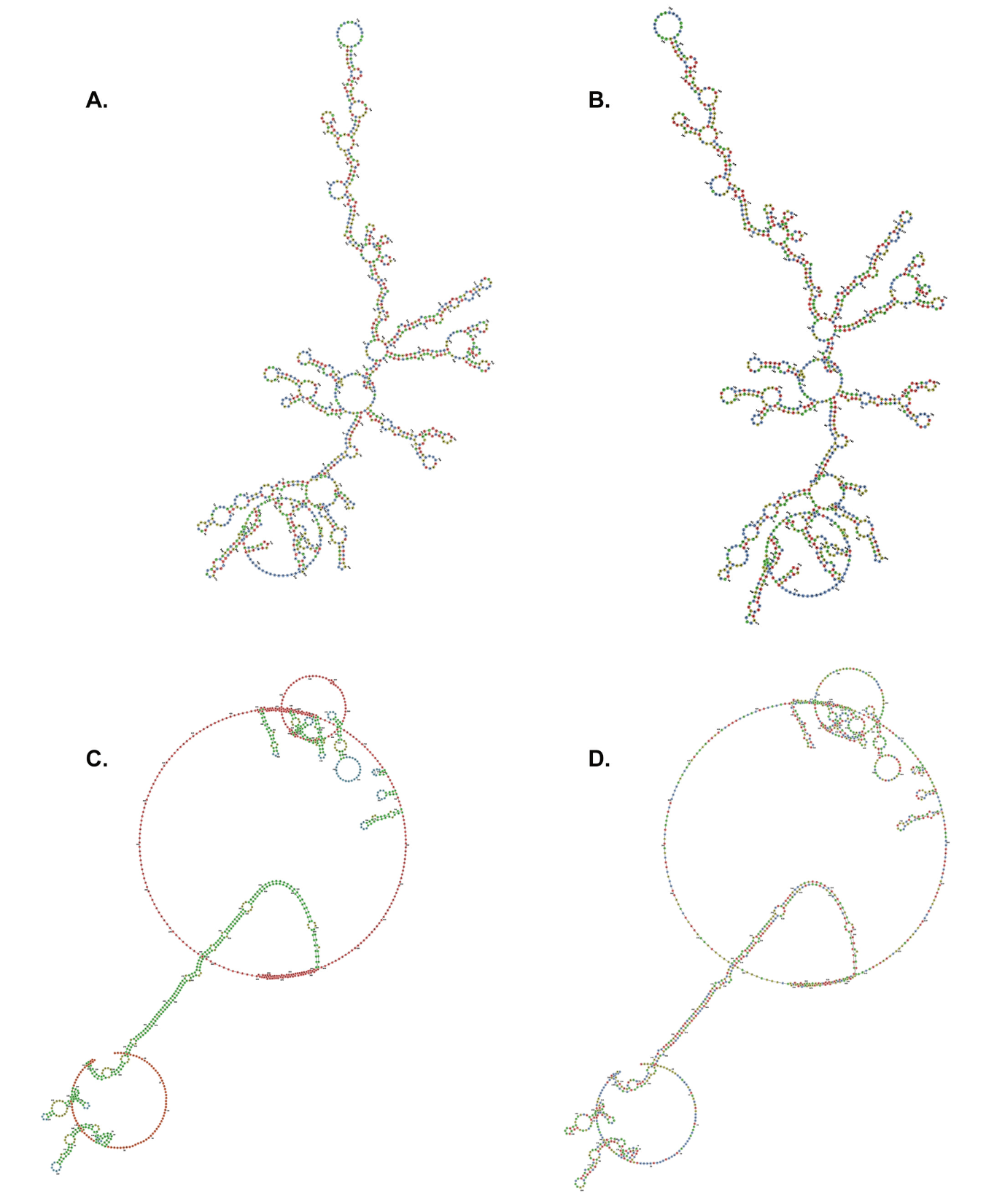


**S3 Fig:** **RNA Secondary structure** of A. wildtype LDLR gene (c.103C>T region). B. mutant LDLR gene (c.103C>T region). C. wildtype LDLR gene (c.2416dupG region). And D. Mutant LDLR gene (c.2416dupG region).

|  | **Patient ID** | **LDL** | **HDL** | **Cholesterol** | **Triglycerides** | **HB1C %** | **Creatinine** | **ALT** | **TSH** |
| --- | --- | --- | --- | --- | --- | --- | --- | --- | --- |
|  |  | **(mg/dl)** | **(mg/dl)** | **(mg/dl)** | **(mg/dl)** |  | **(mg/dl)** | **(U/L)** | **(uIU/ml)** |
| **Family A** | III-11 | 360 | 49 | 445 | 138 | 5.4 | 0.75 | 11 | 1.27 |
|  | II-2 | 107 | 41 | 170 | 97 | 9.1 | 0.77 | 12 | 2.28 |
|  | IV-15 | 90 | 54 | 155 | 53 | 4.9 | 0.67 | 9 | 1.18 |
|  | IV-16 | 296 | 70 | 383 | 86 | 5.1 | 0.75 | 9 | 1.49 |
|  | IV-17 | 86 | 71 | 165 | 44 | 5.1 | 0.66 | 9 | 1.96 |
|  | IV-18 | 85 | 60 | 156 | 59 | 5.2 | 0.65 | 7 | 1.56 |
|  | III-2 | 409 | 71 | 495 | 74 | 5.2 | 0.69 | 10 | 2.18 |
|  | IV-2 | 134 | 44 | 213 | 174 | 5.3 | 1 | 33 | 2.39 |
|  | IV-3 | 386 | 52 | 453 | 76 | 4.9 | 0.74 | 11 | 1.02 |
|  | IV-4 | 369 | 26 | 435 | 202 | 4.5 | 0.71 | 15 | 1.7 |
|  | IV-5 | 143 | 42 | 205 | 102 | 5.7 | 0.95 | 33 | 1.36 |
|  | IV-6 | 295 | 34 | 354 | 125 | 5.1 | 0.68 | 14 | 1.56 |
| **Family B** | II-14 | 286 | 30 | 332 | 90 | 5.4 | 0.65 | 7 | 1.14 |
|  | I-1 | 71 | 46 | 131 | 72 | 5.9 | 0.87 | 10 | 0.81 |
|  | II-1 | 88 | 57 | 178 | 164 | 5 | 0.87 | 9 | 1.1 |
|  | II-7 | 113 | 65 | 193 | 73 | 8.4 | 0.6 | 9 | 0.7 |
|  | II-9 | 137 | 59 | 210 | 70 | 5 | 0.86 | 7 | 1.01 |
|  | II-11 | 516 | 23 | 579 | 198 | < 4.0 | 0.79 | 8 | 1.06 |
|  | II-12 | 143 | 75 | 238 | 98 | 4.9 | 0.86 | 26 | 1.34 |
|  | II-15 | 118 | 66 | 191 | 35 | 4.9 | 0.65 | 10 | 0.94 |

**Supplementary Table 1:** Lipid profile and other biochemical tests (lipid profile and another test) for enrolled individuals.
